# The scaffold protein IQGAP1 promotes reorientation of epithelial cell polarity at the two‐cell stage for cystogenesis

**DOI:** 10.1111/gtc.13169

**Published:** 2024-10-08

**Authors:** Michihiro Horikawa, Junya Hayase, Sachiko Kamakura, Akira Kohda, Masafumi Nakamura, Hideki Sumimoto

**Affiliations:** ^1^ Department of Biochemistry Kyushu University Graduate School of Medical Sciences Fukuoka Japan; ^2^ Department of Surgery and Oncology Kyushu University Graduate School of Medical Sciences Fukuoka Japan

**Keywords:** apical protein endocytosis, epithelial cell polarity, IQGAP1, MDCK cells, polarity reorientation, Rac1, Tiam1, two‐cell stage

## Abstract

A single epithelial cell embedded in extracellular matrix (ECM) can proliferate to form an apical lumen‐harboring cyst, whose formation is a fundamental step in epithelial organ development. At an early two‐cell stage after cell division, the cell doublet typically displays “inverted” polarity, with apical and basolateral proteins being located to the ECM‐facing and cell–cell‐contacting plasma membranes, respectively. Correct cystogenesis requires polarity reorientation, a process containing apical protein endocytosis from the ECM‐abutting periphery and subsequent apical vesicle delivery to a cell–cell contact site for lumen formation. Here, we show that downstream of the ECM‐signal‐transducer β1‐integrin, Rac1, and its effector IQGAP1 promote apical protein endocytosis, contributing to polarity reorientation of mammalian epithelial Madin‐Darby canine kidney (MDCK) cells at a later two‐cell stage in three‐dimensional culture. Rac1–GTP facilitates IQGAP1 interaction with the Rac‐specific activator Tiam1, which also contributes to the endocytosis and enhances the effect of IQGAP1. These findings suggest that Tiam1 and IQGAP1 form a positive feedback loop to activate Rac1. With Rac1–GTP, IQGAP1 also binds to AP2α, an adaptor protein subunit for clathrin‐mediated endocytosis; depletion of the AP2 complex impairs apical protein endocytosis in MDCK doublets. Thus, Rac1 likely participates in polarity reorientation at the two‐cell stage via its interaction with IQGAP1.

## Graphical abstract

After the first mitosis of a single epithelial cell embedded in extracellular matrix (ECM), the cell doublet typically displays “inverted” polarity, and correct cystogenesis requires polarity reorientation, a process containing apical protein endocytosis from the ECM‐abutting periphery and apical vesicle exocytosis to the cell–cell contact site. In apical protein endocytosis, ECM‐induced clustering of β1‐integrin appears to activate Rac1 provably via Tiam1, a Rac‐specific guanine nucleotide exchange factor. Rac1–GTP formed interacts with its effector IQGAP1, and the Rac1‐bound IQGAP1 then associates with the AP2 complex, leading to apical protein endocytosis.

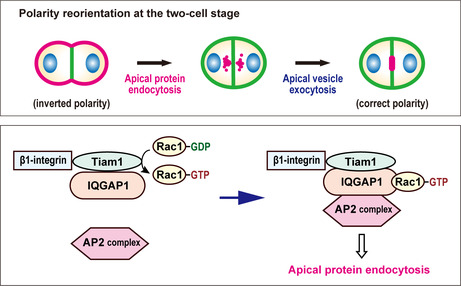

## 1 INTRODUCTION

Tubes of epithelial cells enclosing a central lumen are a fundamental unit of organ design (Bernascone et al., 2017; Lubarsky & Krasnow, 2003). Tubular structures, such as alveoli and cysts, share a common organization in which the apical surface of epithelial cells lines the central lumen and the lateral and basal membranes are attached to neighboring cells and an extracellular matrix (ECM), respectively. The position of the apical lumen is determined by the orientation of the apico‐basal polarity axis in each epithelial cell. In vitro cystogenesis by immortalized cells, including Madin‐Darby canine kidney (MDCK) epithelial cells, is a useful model of epithelial morphogenesis and organogenesis (Buckley & St Johnston, 2022) as well as the pathology of polycystic kidney disease (Dixon & Woodward, 2018).

**FIGURE 1:**
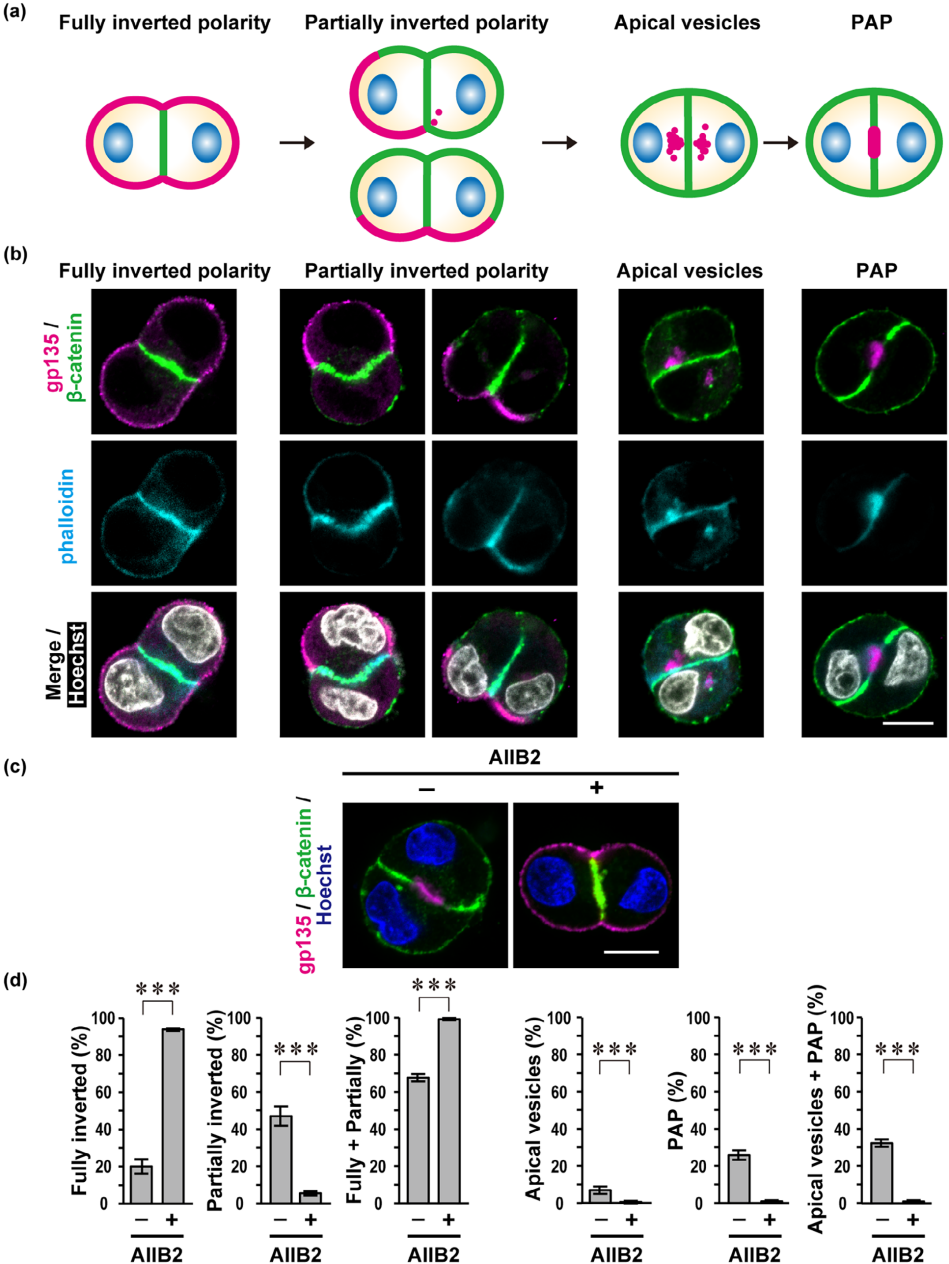
β1‐integrin is required for apical protein endocytosis during polarity reorientation at the two‐cell stage of three‐dimensional (3D)‐cultured Madin‐Darby canine kidney (MDCK) cells. (a) Schematic representation of a transition of the distribution of apical (magenta) and basolateral (green) membrane proteins at the two‐cell stage of 3D‐cultured MDCK cells. Blue ellipses indicate nuclei. PAP, pre‐apical patch. (b) Representative confocal images of cell doublets with fully‐inverted polarity, partially‐inverted polarity, apical vesicles, or a PAP. Single MDCK cells were grown for 24 h in Matrigel, followed by fixation and staining with phalloidin, Hoechst, and antibodies against gp135 (apical marker) and β‐catenin (basolateral marker). (c) Representative confocal images of MDCK doublets treated with the monoclonal antibody against β1‐integrin (AIIB2) to inhibit Matrigel‐induced integrin activation. Single cells were cultured for 24 h in Matrigel with (+) or without (−) AIIB2 and visualized with the indicated antibodies and Hoechst. (d) Quantification of cell doublets with fully‐inverted polarity, partially‐inverted polarity, apical vesicles, or a PAP in 3D culture of MDCK cells treated as in (c). The sum of the fully‐ and partially‐inverted polarity (Fully + Partially) and the sum of the apical vesicles and PAP (Apical vesicles + PAP) are also shown. Values are means ± SD from four independent experiments (*n* ≥ 75 doublets/experiment). ***p < .001 (Tukey–Kramer test). The scale bars represent 10 μm.

Single MDCK cells are unpolarized without external cues such as ECM (Bryant et al., 2014; Mrozowska & Fukuda, 2016). When cultured in three‐dimensional (3D) ECM, especially in laminin‐rich Matrigel (Matlin et al., 2017), MDCK cells undergo a series of stereotyped, but not always synchronous, morphogenetic steps from a single cell to a cyst with a single, central lumen that is surrounded by cells with correct apico‐basal polarity (Buckley & St Johnston, 2022; Román‐Fernández & Bryant, 2016). At an early two‐cell stage after the first cell division, a substantial portion of the cell doublets display “inverted” polarity in that apical transmembrane proteins are located to the ECM‐abutting periphery, whereas basolateral proteins are present at the cell–cell contact region between the doublet (Bryant et al., 2010, 2014; Mrozowska & Fukuda, 2016) (see Figure 1a). The polarity is thus required to be reoriented for proper development of an apical lumen during cystogenesis. In polarity reorientation at the two‐cell stage, apical proteins are endocytosed from the cell–ECM interface as apical vesicles, which are subsequently delivered to the cell–cell contact region to generate pre‐apical patch (PAP), representing an early stage of apico‐basal polarity. The doublet with correct polarity proliferates to develop a cyst with a lumen originated from the PAP (Bryant et al., 2010; Ferrari et al., 2008).

In the step of apical vesicle delivery from the cytoplasm to the cell–cell contact region, the small GTPase Cdc42 plays a crucial role (Bryant et al., 2010). On the other hand, apical protein endocytosis, that is, the initial step of polarity reorientation, is considered to be elicited by detection of ECM via β1‐integrins (Yu et al., 2005). Downstream β1‐integrin signaling, focal‐adhesion kinase (FAK) activates p190RhoGAP, resulting in local hydrolysis of RhoA–GTP to facilitate the endocytic removal of apical proteins (Bryant et al., 2014; Yu et al., 2008). In MDCK cysts grown in type I collagen gels, the small GTPase Rac1, another β1‐integrin effector, is also implicated in polarity reorientation, which is considered to be mediated mainly via promoting basolateral assembly of the ECM component laminin released by MDCK cells (O'Brien et al., 2001; Yu et al., 2005). However, Rac1 is still crucial in MDCK cells cultured in the laminin‐rich Matrigel (Monteleon et al., 2012; Myllymäki et al., 2011). Thus, the molecular mechanism for Rac1‐promoted polarity reorientation has not been fully understood.

In the present study, we show that Rac1 and its effector IQGAP1, a scaffold protein that is known to bind to β1‐integrins (Thines et al., 2023), promote apical protein endocytosis from the cell–ECM interface, contributing to reorientation of apico‐basal polarity in MDCK cells at the two‐cell stage. In the presence of Rac1–GTP, IQGAP1 interacts with Tiam1, a guanine nucleotide exchange factor (GEF) that can specifically activate Rac (Karnoub et al., 2001); Tiam1 also participates in apical protein endocytosis and is able to further facilitate the effect of IQGAP1 on MDCK cells. Thus, Rac1 is likely activated via a positive feedback loop composed of its activator Tiam1 and its effector IQGAP1. In the presence of Rac1–GTP, IQGAP1 also binds to AP2α, a subunit of the adaptor protein 2 (AP2) complex for clathrin‐mediated endocytosis from the plasma membrane (Beacham et al., 2019; Mettlen et al., 2018). Depletion of the AP2 complex impairs apical protein endocytosis in MDCK cells at the two‐cell stage. These findings indicate that the Rac1 effector IQGAP1 facilitates the endocytic removal of apical proteins from the cell–ECM interface, possibly by interacting with the AP2 complex.

## 2 RESULTS

### 2.1 β1‐integrin is required for apical protein endocytosis during polarity reorientation at the two‐cell stage of 3D‐cultured MDCK cells

After the first division of MDCK cells in 3D Matrigel culture, the doublets typically display “inverted” cell polarity with apical proteins locating solely to the ECM‐abutting plasma membrane (Bryant et al., 2010; Bryant et al., 2014; Mrozowska & Fukuda, 2016). Proper lumen formation requires reorientation of the polarity at the two‐cell stage. This reorientation proceeds with the following steps: apical proteins are initially distributed to the cell–ECM interface and then removed by endocytosis to form intracellular apical vesicles; and these apical vesicles are finally delivered from the cytoplasm to the cell–cell contact region to generate PAP, resulting in doublets with “correct” apico‐basal polarity (Figure 1a).

To investigate polarity reorientation at the two‐cell stage, we carefully examined the distribution of apical and basolateral proteins in MDCK cell doublets, which were derived from single cells cultured for 24 h in 3D Matrigel (Figure 1b). Under the conditions, about 60% of the doublets showed “inverted” cell polarity (59%, *n* = 323), in that the basolateral marker β‐catenin localized to the cell–cell contact and the apical transmembrane protein gp135 distributed to the ECM‐abutting periphery (Figure 1b). Among them, doublets with gp135 distributing to more than 75% or less than 75% of the ECM‐facing plasma membrane were defined as those with fully‐inverted polarity (21%, *n* = 323) or with partially‐inverted polarity (39%, *n* = 323), respectively. In a part of doublets as designated “with apical vesicles” (8%, *n* = 323), gp135 was entirely removed from the cell–ECM interface and accumulated in intracellular apical vesicles that were rich in actin filaments but free from β‐catenin. Doublets with reoriented polarity harbored a gp135‐enriched PAP (31%, *n* = 323), a precursor of the apical membrane facing a lumen of the cyst (Bryant et al., 2010; Ferrari et al., 2008). Polarity reorientation of MDCK doublets thus appears to proceed from those “with fully‐inverted polarity” via “with partially‐inverted polarity” and “with apical vesicles” to “with PAP” (Figure 1a,b).

To know the role for ECM‐triggered β1‐integrin activation in polarity reorientation in MDCK doublets, we treated MDCK cells with AIIB2, an anti‐β1‐integrin monoclonal antibody that blocks β1‐integrin activation (Yu et al., 2005). The treatment led to a marked increase in cell doublets with fully‐inverted polarity (93.7%, *n* = 301) (Figure 1c,d), suggesting that apical protein endocytosis is impaired by the blockade of β1‐integrin signaling. Consistent with this, AIIB2 caused a loss of the doublets that were formed after completion of apical protein endocytosis, that is, doublets with apical vesicles (0.3%, *n* = 301) and those with PAP (0.7%, *n* = 301) (Figure 1d). Furthermore, doublets with partially‐inverted polarity, in which apical proteins are vigorously endocytosed, were also significantly decreased by AIIB2 (Figure 1d). These findings indicate that ECM‐directed β1‐integrin activation is required for apical protein endocytosis during polarity reorientation at the two‐cell stage of Matrigel‐embedded MDCK cells, which agrees with the idea that β1‐integrin is not necessary for cell polarization per se but instead reorients cell polarity from initially inverted to lumen‐containing cysts (Bryant et al., 2014).

### 2.2 Rac1 promotes apical protein endocytosis during polarity reorientation at the two‐cell stage in 3D‐cultured MDCK cells

**FIGURE 2:**
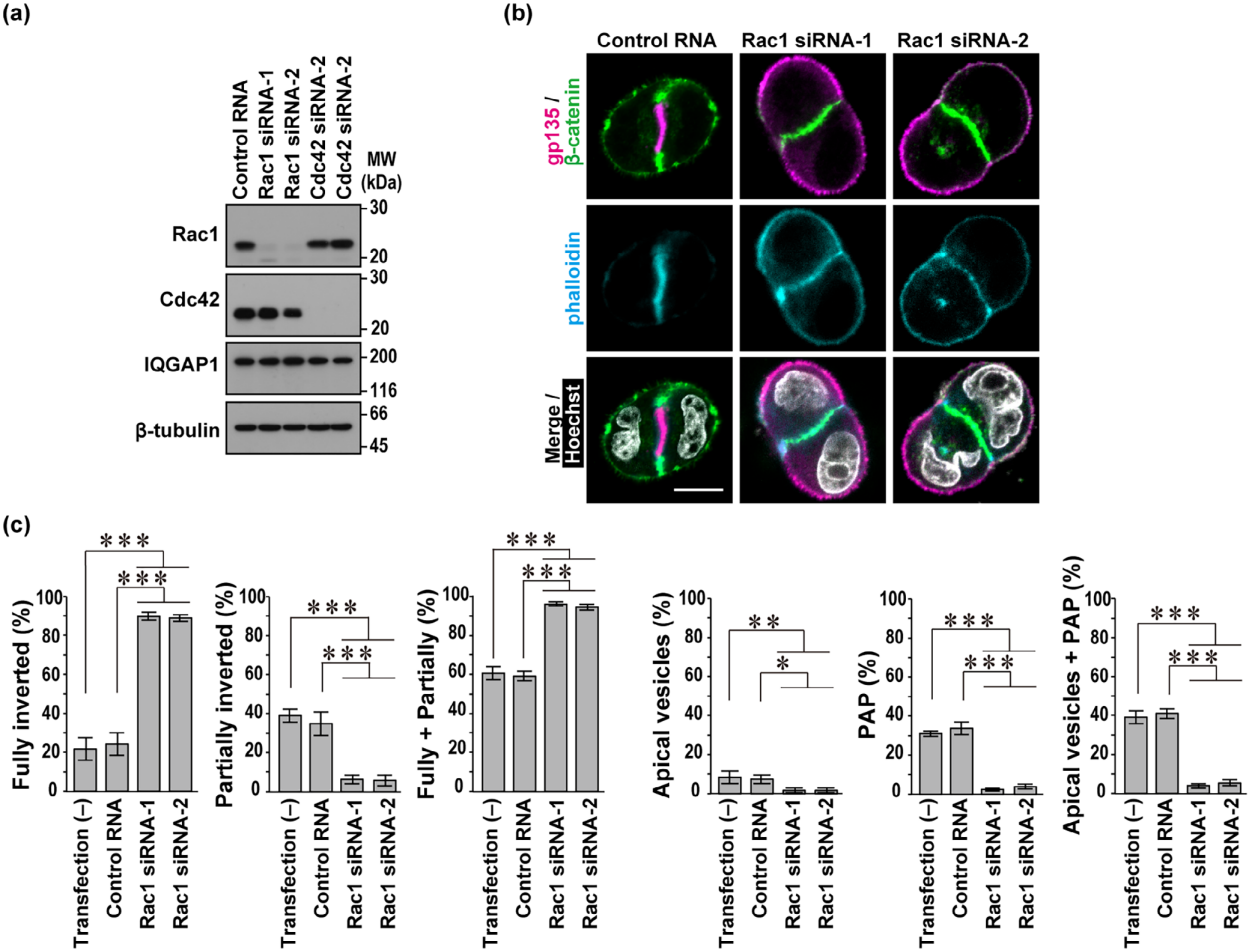
Rac1 promotes apical protein endocytosis during polarity reorientation at the two‐cell stage in three‐dimensional (3D)‐cultured Madin‐Darby canine kidney (MDCK) cells. (a) Immunoblot analysis of the lysate from MDCK cells transfected with negative control RNA, Rac1‐specific small interfering RNAs (siRNA) (Rac1 siRNA‐1 and siRNA‐2), or Cdc42‐specific siRNA (Cdc42 siRNA‐1 and siRNA‐2). Proteins in the lysates were analyzed by immunoblot with antibodies against Rac1, Cdc42, IQGAP1, and β‐tubulin. Positions for marker proteins are indicated in kDa. (b) Representative confocal images of MDCK doublets in 3D culture. Cells transfected with control RNA or Rac1 siRNA were subcultured for 24 h and trypsinized to a single cell suspension; the resultant single cells were cultured for 24 h in Matrigel, followed by staining with the indicated antibodies and Hoechst. The scale bar represents 10 μm. (c) Quantification of cell doublets with fully‐inverted polarity, partially‐inverted polarity, apical vesicles, or a pre‐apical patch (PAP) in 3D culture of MDCK cells treated as in (b). The sum of the fully‐ and partially‐inverted polarity (Fully + Partially) and the sum of the apical vesicles and PAP (Apical vesicles + PAP) are also shown. Values are means ± SD from four independent experiments (*n* ≥ 75 doublets/experiment). *p < .05; **p < .01; and ***p < .001 (Tukey–Kramer test).

Expression of the dominant negative form of Rac1 (T17N) is known to induce the formation of MDCK cell clusters with inverted polarity (Myllymäki et al., 2011; O'Brien et al., 2001). To clarify the role of Rac1 at the two‐cell stage, we depleted Rac1 in MDCK cells by small interfering RNAs (siRNA)‐mediated RNA interference (Figure 2a) and tested its effect on polarity reorientation (Figure 2b,c). Depletion of Rac1 led to a pronounced increase in fully‐inverted doublets with a concomitant decrease in the two‐cell aggregates where endocytic removal of apical proteins from the cell periphery is in progress or completed, such as doublets with partially‐inverted polarity, those with apical vesicles, and those with PAP (Figure 2c). Thus, similar to β1‐integrin (Figure 1d), its downstream effector Rac1 appears to play a crucial role in apical protein endocytosis for polarity reorientation of MDCK cells at the two‐cell stage in Matrigel 3D culture.

### 2.3 Rac1 orients plasma membrane polarity but not intracellular organelle positioning

**FIGURE 3:**
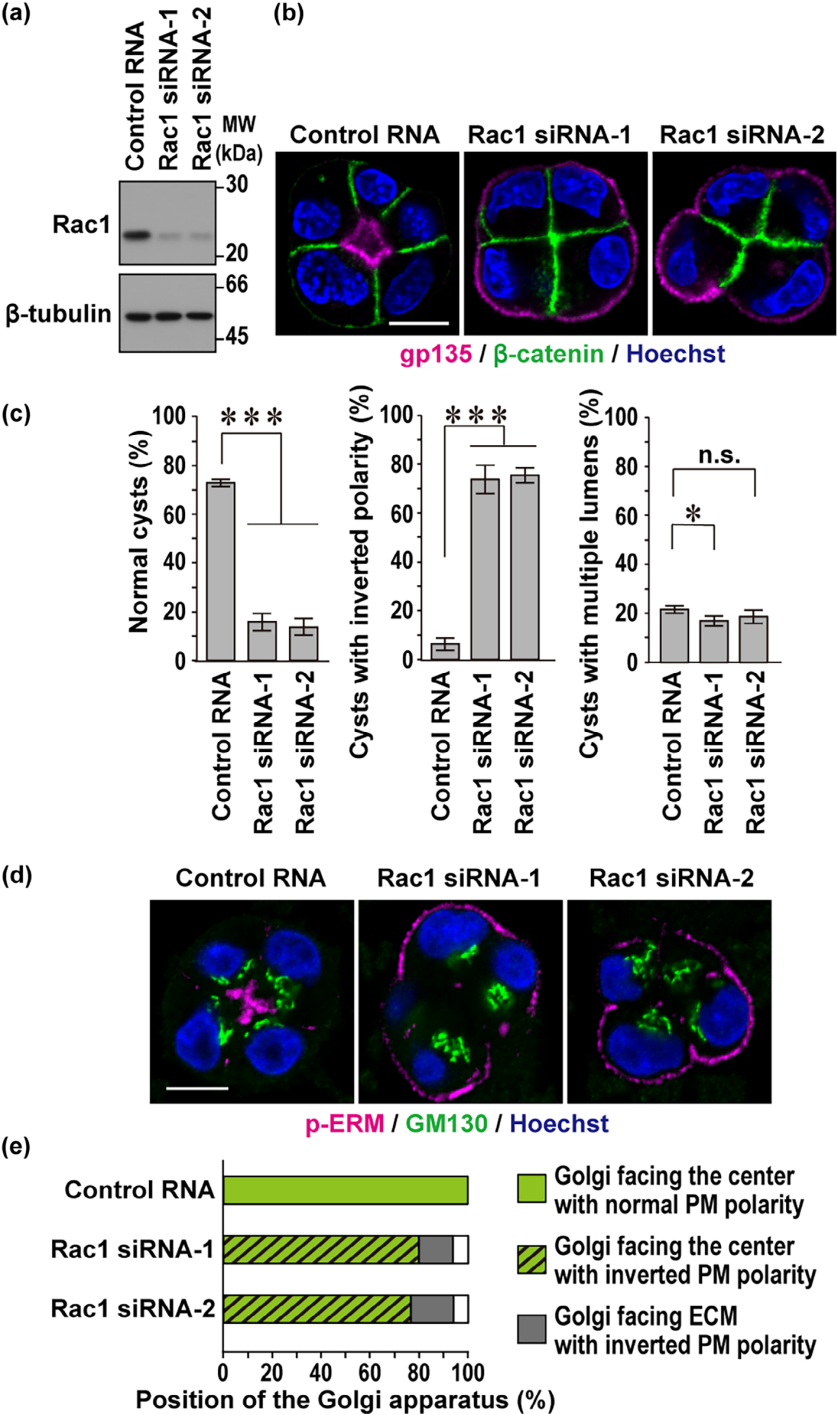
Rac1 orients plasma membrane polarity but not positioning of intracellular organelles. (a) Immunoblot analysis of Rac1 and β‐tubulin expression in Madin‐Darby canine kidney (MDCK) cells transfected with negative control RNA or Rac1‐specific small interfering RNAs (siRNA). Positions for marker proteins are indicated in kDa. (b) Representative confocal images of Rac1‐depleted MDCK cells in three‐dimensional (3D) culture. Cells transfected with the indicated RNA were grown for 48 h in Matrigel and visualized with the indicated antibodies and Hoechst. (c) Quantification of the cyst phenotypes of 3D‐cultured MDCK cells: Normally‐oriented cysts with a solitary lumen (normal cysts), cysts with inverted polarity, or cysts with multiple lumens. Values are means ± SD from four independent experiments (*n* ≥ 75 cysts/experiment). *p < .05; ***p < .001; and n.s., not significant (Tukey–Kramer test). (d) Representative confocal images of Rac1‐depleted MDCK cells in 3D culture. Cysts cultured for 48 h in Matrigel were fixed and stained with the antibodies against GM130 (Golgi marker), phosphorylated ezrin/radixin/moesin (p‐ERM) (apical marker) and Hoechst. (e) The position of the Golgi apparatus relative to the nucleus in 3D‐cultured MDCK cells treated as in (d). Bar graphs indicate quantification of cells in which the Golgi faces the center of the cell cluster with normal plasma membrane (PM) polarity (green open box), the Golgi faces the center of the cluster with inverted PM polarity (green dashed box), or the Golgi faces extracellular matrix (ECM) with inverted PM polarity (gray box). At least 100 cells were counted for each condition. The scale bars represent 10 μm.

To study the role for Rac1 in polarization at later stages, we examined multicellular MDCK cysts, comprising four to eight cells, after Matrigel 3D culture for 48 h, when the Rac1 protein level remained to be fully reduced in Rac1 siRNA‐transfected cells (Figure 3a). Depletion of Rac1 significantly decreased normal (correctly‐oriented) cysts with a single lumen, the surface of which was positive for gp135 and with the basolateral marker β‐catenin localizing to sites of cell–cell contact and to those facing the ECM, without increasing cysts with multiple lumens, which are typical in Cdc42‐depleted MCDK cells (Martin‐Belmonte et al., 2007) (Figure 3b,c). Instead, Rac1 knockdown resulted in a marked increase in cell clusters with inverted polarity, in which gp135 localized at the ECM‐abutting plasma membrane (Figure 3b,c). Thus, the polarity that is not reoriented at the two‐cell stage seems to remain uncorrected at later stages of cystogenesis in the absence of Rac1.

In contrast to misoriented polarity of plasma membrane proteins, positioning of intracellular organelles is not severely perturbed in cysts with inverted orientation induced by Rac1 depletion. Nuclei were localized on the side of the ECM‐abutting plasma membrane in both control and Rac1‐depleted cysts (Figure 3d,e). The Golgi marker protein GM130 was enriched apical to the nucleus in control cysts (100%, *n* = 100) (Figure 3d,e). On the other hand, in 80% of inverted cell clusters of Rac1‐depleted cells (*n* = 102), the Golgi apparatus was retained between the nucleus and the center of the cluster instead of the localization of the apical protein gp135 at the ECM‐abutting periphery (Figure 3d,e). These findings suggest that Rac1 contributes to reorientation of plasma membrane polarity without affecting cytoplasmic organelles, probably via endocytosis of apical proteins from the periphery, the initiation step of which is independent of the Golgi apparatus.

### 2.4 Cdc42 is dispensable for apical protein endocytosis from the cell–ECM interface but crucial for apical vesicle delivery to the cell–cell contact site

**FIGURE 4:**
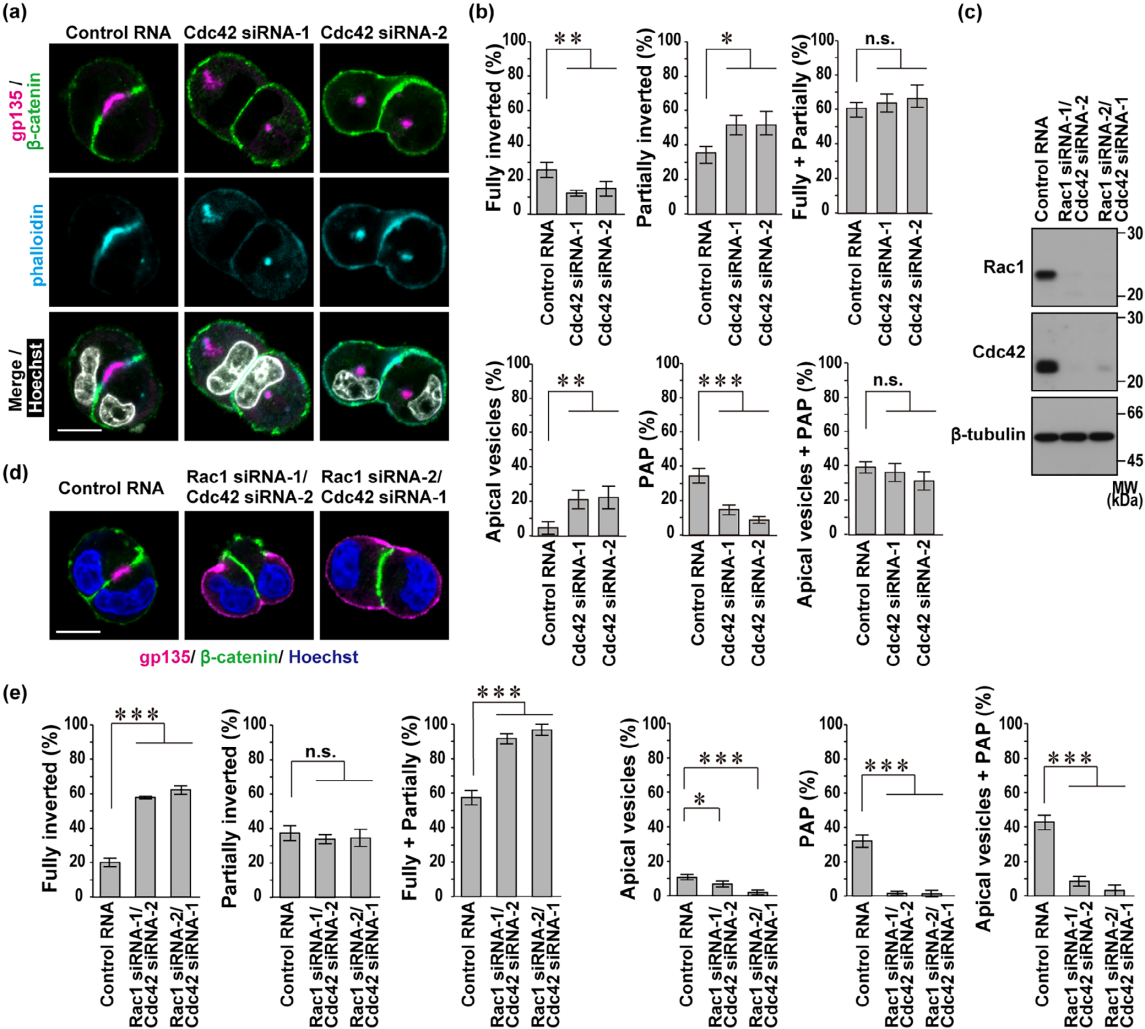
Cdc42 is dispensable for apical protein endocytosis from the cell–extracellular matrix (ECM) interface but crucial for apical vesicle delivery to the cell–cell contact site. (a and d) Representative confocal images of Madin‐Darby canine kidney (MDCK) doublets in three‐dimensional (3D) culture. Cells transfected with the indicated RNA (control RNA, Cdc42‐specific small interfering RNAs [siRNA], and Rac1‐specific siRNA) were cultured for 24 h in Matrigel, followed by staining as indicated. The scale bars represent 10 μm. (b and e) Quantification of cell doublets with fully‐inverted polarity, partially‐inverted polarity, apical vesicles, or a pre‐apical patch (PAP) in 3D culture of MDCK cells transfected with the indicated RNA. The sum of the fully‐ and partially‐inverted polarity (Fully + Partially) and the sum of the apical vesicles and PAP (Apical vesicles + PAP) are also shown. Values are means ± SD from four independent experiments (*n* ≥ 75 doublets/experiment). *p < .05; **p < .01; ***p < .001; and n.s., not significant (Tukey–Kramer test). (c) Immunoblot analysis of Rac1, Cdc42, and β‐tubulin expression in MDCK cells transfected with the indicated RNA. Positions for marker proteins are indicated in kDa.

It is known that Cdc42, regulated by the Rab family GTPases Rab11a and Rab8a, is required for apical vesicle delivery to the cell–cell contact site to initiate proper lumen formation by Matrigel‐embedded MDCK cells (Bryant et al., 2010). To further understand the difference between the two Rho family GTPases Cdc42 and Rac1 in polarity reorientation, we depleted Cdc42 in MDCK cells (Figure 2a) and tested its effect at the two‐cell stage in 3D Matrigel culture. Depletion of Cdc42 markedly increased doublets with apical vesicles, with a concomitant decrease of doublets with PAP, although the sum of the two types of doublets, both in a state after completion of apical protein endocytosis, was not significantly changed (Figure 4a,b). Thus, apical vesicle delivery for PAP formation was impaired in Cdc42‐depleted cells, confirming that Cdc42 is crucial for apical vesicle exocytosis. On the other hand, Cdc42 knockdown did not affect the sum of the fully‐ and partially‐inverted doublets (Figure 4a,b), indicating that Cdc42 is dispensable for apical protein endocytosis at the two‐cell stage.

Taken together with the present findings, in cell polarity reorientation at the two‐cell stage, Rac1 primarily promotes endocytic removal of apical proteins from the cell–ECM interface, leading to accumulation of apical vesicles, which are subsequently delivered to the cell–cell contact region to form PAP in a Cdc42‐dependent manner. The order of action of the two GTPases in polarity reorientation was supported by experiments using MDCK cells in which Rac1 and Cdc42 were doubly knocked down (Figure 4c). The double knockdown of Rac1 and Cdc42 led to a marked increase in fully‐inverted doublets but a significant decrease in apical vesicle‐ and PAP‐containing doublets (Figure 4d,e), which changes are similar to those observed in Rac1 single knockdown cells (Figure 2b,c).

### 2.5 The Rac1‐binding protein IQGAP1 is involved in apical protein endocytosis during polarity reorientation at the two‐cell stage

**FIGURE 5:**
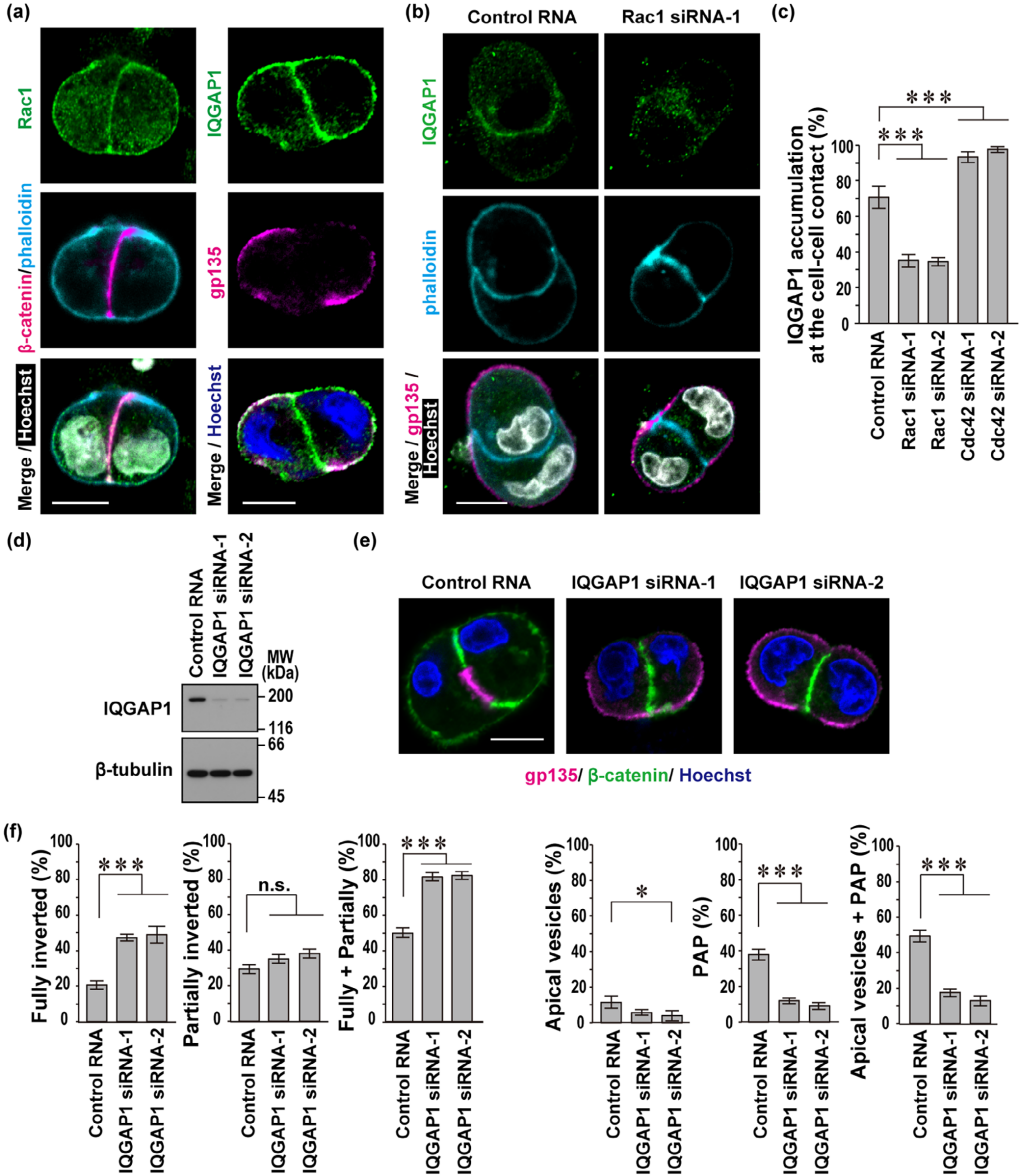
The Rac1‐binding protein IQGAP1 is involved in apical protein endocytosis during polarity reorientation at the two‐cell stage. (a, b, and e) Representative confocal images of Madin‐Darby canine kidney (MDCK) doublets in three‐dimensional (3D) culture. Untransfected cells (a) or cells transfected with the indicated RNA (b and e) were cultured for 24 h in Matrigel, followed by staining with indicated antibodies, phalloidin, and Hoechst. The scale bars represent 10 μm. (c) Quantification of IQGAP1 accumulation at the cell–cell contact of doublets with inverted polarity. Values are means ± SD from four independent experiments (*n* ≥ 30 doublets/experiment). (d) Immunoblot analysis of IQGAP1 and β‐tubulin expression in MDCK cells transfected with negative control RNA or IQGAP1‐specific small interfering RNAs (siRNA) (IQGAP1 siRNA‐1 or siRNA‐2). Positions for marker proteins are indicated in kDa. (f) Quantification of cell doublets with fully‐inverted polarity, partially‐inverted polarity, apical vesicles, or a pre‐apical patch (PAP) in 3D culture of MDCK cells treated as in (e). The sum of the fully‐ and partially‐inverted polarity (Fully + Partially) and the sum of the apical vesicles and PAP (Apical vesicles + PAP) are also shown. Values are means ± SD from four independent experiments (*n* ≥ 75 doublets/experiment). *p < .05; ***p < .001; and n.s., not significant (Tukey–Kramer test).

In a MDCK doublet with inverted polarity, endogenous Rac1 localized to cell–cell and cell–ECM contacts (Figure 5a), which is consistent with the localization of ectopically‐expressed, GFP‐fused Rac1 in 3D‐cultured MDCK cells (Martin‐Belmonte et al., 2007). To know Rac1 effectors involved in polarity reorientation, we expressed the constitutively active form of Rac1 (Q61L) in MDCK cells and analyzed its co‐precipitated proteins by liquid chromatography–tandem mass spectrometry (LC–MS/MS) (see Section 4). The analysis demonstrated that IQGAP1 was a major Rac1‐binding protein in MDCK cells. Similar to Rac1, endogenous IQGAP1 localized to cell–cell and cell–ECM contacts in a MDCK doublet with inverted polarity (Figure 5a). The localization of IQGAP1 was impaired by depletion of Rac1 but not by that of Cdc42 (Figure 5b,c), suggesting that IQGAP1 may function with Rac1 in apical protein endocytosis from the cell periphery. To test this possibility, we examined cell polarization in IQGAP1‐depleted MDCK cells (Figure 5d). Depletion of IQGAP1 led to a significant increase in fully‐inverted doublets and a decrease in apical vesicle‐ and PAP‐containing doublets (Figure 5e,f). Thus, IQGAP1 is likely involved in endocytic removal of apical membrane proteins from the cell periphery.

### 2.6 The Rac activator Tiam1 interacts with IQGAP1 and promotes apical protein endocytosis at the two‐cell stage

**FIGURE 6:**
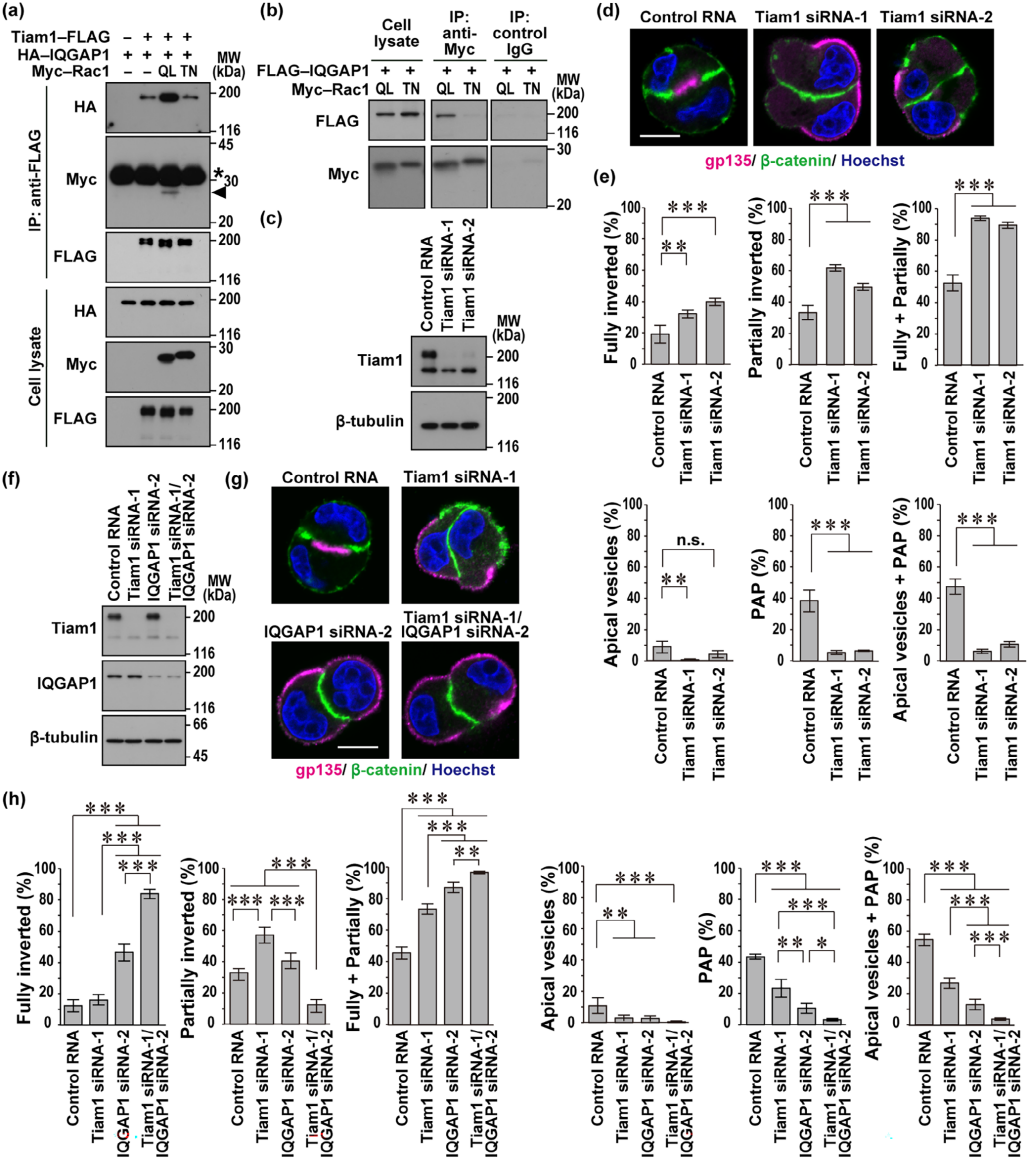
The Rac activator Tiam1 interacts with IQGAP1 and promotes apical protein endocytosis at the two‐cell stage of three‐dimensional (3D)‐cultured Madin‐Darby canine kidney (MDCK) cells. (a) IQGAP1 interacts with Tiam1. FLAG–Tiam1 and HA–IQGAP1 were co‐expressed in HEK293 cells with an empty vector (−), Myc–Rac1 (Q61L), or Myc–Rac1 (T17N). Proteins in the cell lysate (cell lysate) were immunoprecipitated (IP) with the anti‐FLAG antibody, followed by immunoblot analysis with the indicated antibodies. Positions for marker proteins are indicated in kDa. The arrowhead shows the band of Myc–Rac1 (Q61L) co‐precipitated with Tiam1–FLAG. The asterisk indicates the band of the light chain of IgG used for immunoprecipitation. (b) IQGAP1 interacts with Rac1 (Q61L) but not with Rac1 (T17N). Proteins in the lysate of HEK293 cells expressing the indicated proteins (cell lysate) were IP with the anti‐FLAG antibody or control IgG, and then analyzed by immunoblot with the indicated antibodies. (c and f) Immunoblot analysis of MDCK cells transfected with the indicated RNA. (d and g) Representative confocal images of MDCK doublets in 3D culture. Cells transfected with the indicated RNA (control RNA, Tiam1‐specific small interfering RNAs (siRNA), and/or IQGAP1‐specific siRNA) were cultured for 24 h in Matrigel, followed by staining with Hoechst and antibodies against gp135 and β‐catenin. The scale bars represent 10 μm. (e and h) Quantification of cell doublets with fully‐inverted polarity, partially‐inverted polarity, apical vesicles, or a pre‐apical patch (PAP) in 3D culture of MDCK cells. The sum of the fully‐ and partially‐inverted polarity (Fully + Partially) and the sum of the apical vesicles and PAP (Apical vesicles + PAP) are also shown. Values are means ± SD from four independent experiments (*n* ≥ 75 doublets/experiment). *p < .05; **p < .01; ***p < .001; and n.s., not significant (Tukey–Kramer test).

It has been reported that IQGAP1 forms a multi‐protein complex containing Tiam1, a Rac‐specific GEF, in human pulmonary artery endothelial cells (Usatyuk et al., 2009). To investigate the interaction between the Rac activator Tiam1 and the Rac effector IQGAP, we expressed Tiam1–FLAG and HA–IQGAP1 in HEK293 cells and analyzed proteins precipitated with the anti‐FLAG antibody by immunoblot. As shown in Figure 6a, HA–IQGAP1 indeed interacted with Tiam1–FLAG. Intriguingly, the interaction was enhanced by the co‐expression of Myc–Rac1 (Q61L), a constitutively GTP‐bound form of Rac1 (Figure 6a). On the other hand, the co‐expression of Myc–Rac1 (T17N), a dominant negative (GDP‐bound) form of Rac1, did not affect the interaction between IQGAP1 and Tiam1 (Figure 6a). In addition, Rac1 (Q61L), capable of binding to IQGAP1 (Figure 6b), was co‐precipitated with Tiam1–FLAG (Figure 6a), demonstrating the formation of a ternary complex containing Rac1–GTP, IQGAP1, and Tiam1.

Because the present findings show that both Rac1 and IQGAP1 in the ternary complex promote apical membrane endocytosis (Figures 2 and 5), we examined the role of Tiam1, another component of the complex, in polarity reorientation at the two‐cell stage. When MDCK cells were depleted of Tiam1 (Figure 6c), endocytic removal of gp135 from the cell–ECM interface was impaired, as indicated by a significant increase in fully‐ and partially‐inverted doublets with a concomitant decrease in apical vesicle‐ and PAP‐containing doublets (Figure 6d,e). Thus, Tiam1 seems to participate in endocytic removal of apical membrane proteins from the cell periphery.

Compared with the consequence of Rac1 depletion (Figure 2c), knockdown of either IQGAP1 (Figure 5f) or Tiam1 (Figure 6e) appears to exert a milder impact on polarity reorientation. Hence, we next doubly knocked down IQGAP1 and Tiam1 in MDCK cells (Figure 6f) and tested its effect on apical protein endocytosis at the two‐cell stage (Figure 6g,h). The double knockdown of IQGAP1 and Tiam1 resulted in a marked increase of fully‐inverted cell doublets and a significant decrease of partially‐inverted doublets (Figure 6h) to the same extent as that induced by Rac1 knockdown (Figure 2c). Consistent with this, apical vesicle‐ or PAP‐containing doublets were almost completely lost (Figure 6h), as observed in Rac1‐knockdown cells (Figure 2c). These findings indicate that IQGAP1 and Tiam1, forming a ternary complex with Rac1, function together in apical protein endocytosis in polarity reorientation.

### 2.7 IQGAP1 interacts with the AP2 complex, which participates in apical protein endocytosis at the two‐cell stage

The present study shows that a Rac1–Tiam1–IQGAP1 complex promotes polarity reorientation by inducing apical protein endocytosis at the two‐cell stage. To find a link between the complex and endocytosis, we performed LC–MS/MS analysis of proteins co‐precipitated with FLAG‐tagged IQGAP1 in HEK293 cells (see Section 4). The analysis identified AP2α as a candidate for IQGAP1‐binding proteins. AP2α is the α subunit of the AP2 complex, a tetramer that is essential for clathrin‐dependent endocytosis (Beacham et al., 2019; Mettlen et al., 2018).

**FIGURE 7:**
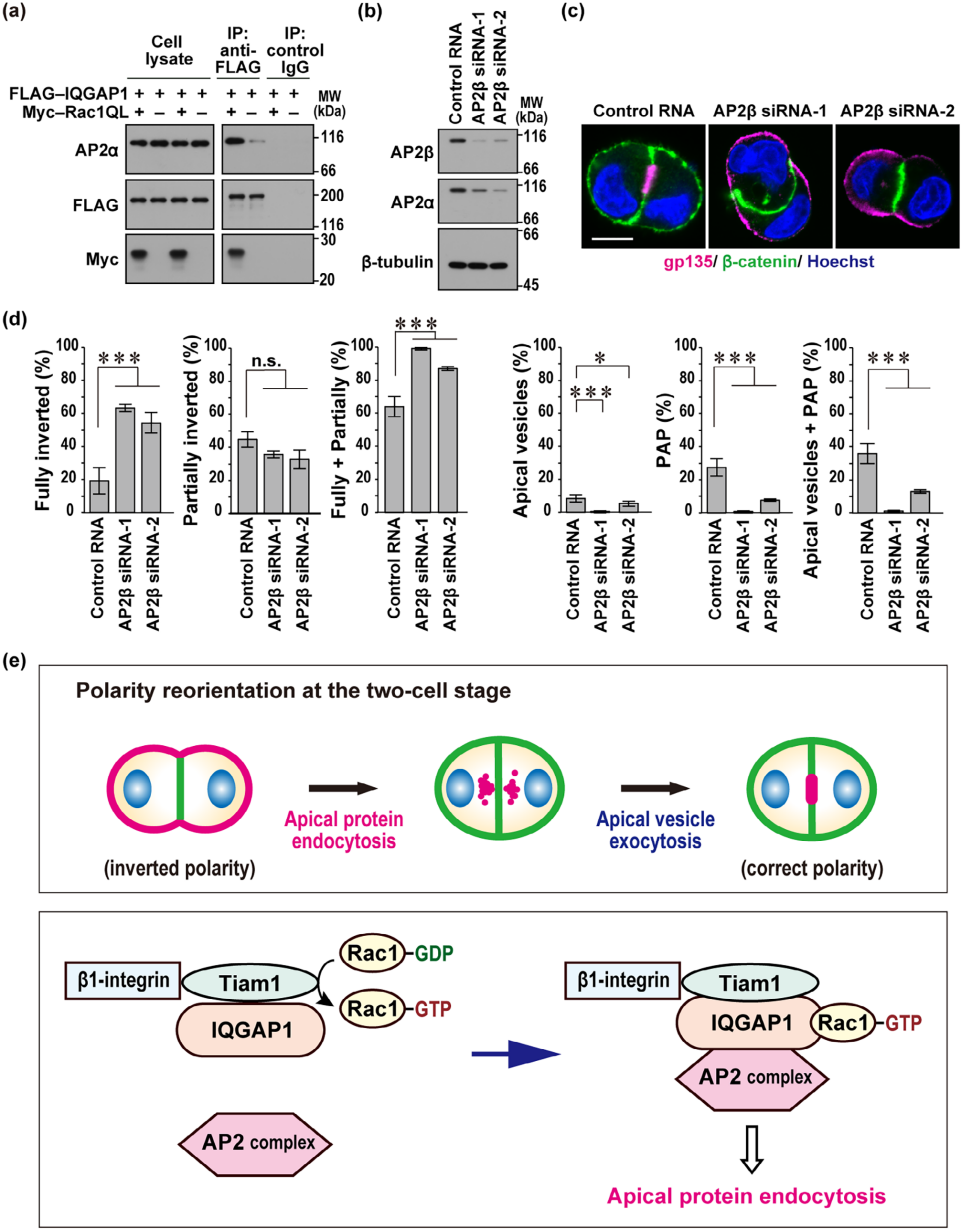
IQGAP1 interacts with the AP2 complex, which participates in apical protein endocytosis at the two‐cell stage of three‐dimensional (3D)‐cultured Madin‐Darby canine kidney (MDCK) cells. (a) Interaction between endogenous AP2α and exogenously expressed FLAG–IQGAP1. FLAG–IQGAP1 and Myc–Rac1 (Q61L) were expressed in HEK293 cells, and proteins in the cell lysate were immunoprecipitated (IP) with the anti‐FLAG antibody or control IgG, followed by immunoblot analysis with the indicated antibodies. (b) Immunoblot analysis for α‐ and β1‐subunits of AP2. Proteins in the lysate of MDCK cells transfected with negative control RNA or AP2B1‐specific small interfering RNAs (siRNA) (AP2 siRNA‐1 or siRNA‐2) were analyzed by immunoblot with the indicated antibodies. Positions for marker proteins are indicated in kDa. (c) Representative confocal images of MDCK doublets in 3D culture. Cells transfected with the indicated RNA were cultured for 24 h in Matrigel, followed by staining with Hoechst and antibodies against gp135 and β‐catenin. The scale bar represents 10 μm. (d) Quantification of cell doublets with fully‐inverted polarity, partially‐inverted polarity, apical vesicles, or a pre‐apical patch (PAP) in 3D culture of MDCK cells. The sum of the fully‐ and partially‐inverted polarity (Fully + Partially) and the sum of the apical vesicles and PAP (Apical vesicles + PAP) are also shown. Values are means ± SD from four independent experiments (*n* ≥ 75 doublets/experiment). *p < .05; ***p < .001; and n.s., not significant (Tukey–Kramer test). (e) A schematic representation for polarity reorientation at the two‐cell stage. After the first mitosis of a single epithelial cell embedded in extracellular matrix (ECM), the cell doublet typically displays “inverted” polarity, and correct cystogenesis requires polarity reorientation, a process containing apical protein endocytosis from the ECM‐abutting periphery and apical vesicle exocytosis to the cell–cell contact site (upper panel). A model of interactions that control apical protein endocytosis at the two‐cell stage for polarity reorientation (lower panel). ECM‐induced clustering of β1‐integrin in epithelial cells leads to activation of Rac1 provably via Tiam1, a Rac‐specific GEF. Due to Tiam1–IQGAP1 interaction, Rac1–GTP formed is easily accessible to its effector IQGAP1. Rac1‐bound IQGAP1 associates with the AP2 complex, thereby inducing apical protein endocytosis, an initial event of polarity reorientation for correct cystogenesis.

For confirmation of the interaction between IQGAP1 and AP2α, FLAG–IQGAP1 was expressed in HEK293 cells, and its interacting proteins were subjected to immunoblot analysis using the anti‐AP2α antibody. As shown in Figure 7a, FLAG–IQGAP1 weakly but significantly interacted with endogenous AP2α. Interestingly, the interaction was strongly enhanced by the presence of Rac1 (Q61L), a GTP‐binding form of Rac1. Thus, it seems likely that Rac1‐induced conformational change renders IQGAP1 in a state fully accessible to AP2α.

To study the role of the AP2 complex in apical protein endocytosis at the two‐cell stage, we knocked down the β subunit of the AP2 complex (AP2β) in MDCK cells (Figure 7b). In AP2β‐depleted cells, the protein level of the α subunit (AP2α) was also substantially reduced (Figure 7b). As shown in Figure 7c,d, depletion of the AP2 complex led to a marked increase of fully‐inverted doublets and a significant decrease in apical vesicle‐ and PAP‐containing doublets. These findings indicate that apical membrane proteins are internalized via AP2‐mediated endocytosis at the two‐cell stage. The AP2‐complex is likely to be recruited by IQGAP1 in a manner dependent on Rac1–GTP, which may be initially formed via the ECM‐driven β1‐integrin signaling and reinforced via the Tiam1–IQGAP1 module.

## 3 DISCUSSION

At an early two‐cell stage after the first cell division during MDCK cystogenesis, the cell doublet typically displays “inverted” polarity, which is required to be reoriented for proper lumen development. The reorientation is triggered by ECM‐directed β1‐integrin activation, which induces the following events: endocytic removal of apical proteins from the cell–ECM interface for apical vesicle formation; and exocytic delivery of apical vesicles from the cytoplasm to the cell–cell contact region for PAP development (Bryant et al., 2010, 2014). In apical vesicle exocytosis at the two‐cell stage, Cdc42 plays a crucial role (Bryant et al., 2010), as confirmed here (Figure 4). In the present study, we show that Rac1, the Rac1 activator Tiam1, and the Rac1 effector IQGAP1 promote apical protein endocytosis, the initial step for polarity reorientation at the two‐cell stage (Figures 2, 5, and 6). Rac1–GTP enhances not only IQGAP1 recruitment to the cell periphery (Figure 5) but also Tiam1–IQGAP1 interaction (Figure 6), implying the important role of the Tiam1–IQGAP1 module in activation of Rac1. Furthermore, IQGAP1 likely links active Rac1 to the AP2 complex, which promotes endocytic removal of apical proteins at the two‐cell stage (Figure 7).

In the present analysis of polarity reorientation at the two‐cell stage, apical protein endocytosis is not (or only slightly) induced in cell doublets with completely inverted polarity, and the endocytosis substantially proceeds in those with partially‐inverted polarity. Blockade of β1‐integrin or depletion of Rac1 each leads to a complete inhibition of polarity reorientation: cells in both cases remain to be fully polarized but with an inverted orientation, as characterized by a marked increase of fully‐inverted doublets and a significant decrease of partially‐inverted ones (Figures 1d and 2c). Polarity reorientation is significantly but not completely inhibited in Tiam1‐ and IQGAP1‐depleted cells. IQGAP1 knockdown leads to a moderate increase of cell doublets with fully‐inverted polarity without decreasing those with partially‐inverted polarity (Figures 5f and 6h), whereas only partially‐inverted doublets, but not completely inverted ones, are increased by Tiam1 depletion (Figure 6h). Thus, the effect of Tiam1 depletion seems to be weaker than that of IQGAP1 knockdown under the present conditions (Figure 6h). Interestingly, compared with single depletion of Tiam1 or IQGAP1, double knockdown of these proteins severely inhibits apical protein endocytosis, as indicated by a marked increase of fully‐inverted cell doublets and a significant decrease of partially‐inverted ones (Figure 6h). This finding supports the idea that Tiam1 and IQGAP1 cooperatively function in apical protein endocytosis (Figure 7e). The endocytosis is also impaired by knockdown of AP2β (Figure 7d), a component of the AP2 complex that interacts with IQGAP1. In contrast to Rac1, Cdc42 is not involved in apical protein endocytosis; cell doublets with inverted polarity are not increased in Cdc42‐depleted cells (Figure 4b). On the other hand, Cdc42 is crucial for apical vesicle exocytosis, a process following apical protein endocytosis in polarity reorientation, as Cdc42 knockdown facilitates retention of apical vesicles in the cytoplasm and thus prevents PAP formation (Figure 4b).

Taken together with the present findings, apical protein endocytosis from the cell–ECM interface, an initial event required for polarity reorientation at the two‐cell stage, is likely controlled via the following mechanism (Figure 7e). ECM‐induced clustering of β1‐integrin appears to activate Rac1, that is, the formation of Rac1–GTP, provably via Tiam1, a Rac‐specific GEF that interacts with IQGAP1 (Figure 6a). Due to the interaction, Rac1–GTP formed is capable of easily binding to its effector IQGAP1, and the binding in turn induces association of IQGAP1 with the AP2 complex for apical protein endocytosis (Figure 7e).

Rac1 and the Tiam1–IQGAP1 module likely form a novel positive feedback loop downstream of β1‐integrin. The interaction between the Rac1 activator Tiam1 and the Rac1 effector IQGAP1 (Figure 6) allows Tiam1‐produced Rac1–GTP to easily access IQGAP1. Importantly, Rac1–GTP markedly enhances Tiam1–IQGAP1 interaction (Figure 6), which further reinforces formation of a complex of Rac1–GTP with its effector IQGAP1. In this context, it seems interesting that Tiam1 but not P‐Rex1, another Rac‐specific GEF, efficiently facilitates the interaction of wild‐type Rac1 with IQGAP1 (Marei et al., 2016), supporting the idea that IQGAP1 specifically interacts with Tiam1 to further activate Rac1. Although IQGAP1 is known to interact with not only Rac1–GTP but also Cdc42–GTP (Thines et al., 2023), the Tiam1‐containing module is not expected to induce Cdc42–IQGAP1 interaction because Tiam1 is incapable of effectively activating Cdc42 (Karnoub et al., 2001). Indeed, as shown here, Cdc42 neither promotes IQGAP1 localization in cell doublets with inverted polarity (Figure 5) nor plays a major role in apical protein endocytosis (Figure 4), a process that requires Tiam1 and IQGAP1 (Figures 5 and 6).

The role of Rac1 in polarity reorientation has been studied using MDCK cells cultured in type I collagen gels (O'Brien et al., 2001; Yu et al., 2005, 2008). Expression of dominant negative Rac1 (T17N) leads to a striking inversion of apical polarity, which is rescued by exogenous laminin (O'Brien et al., 2001); β1‐integrin‐directed activation of Rac1 indeed induces basolateral assembly of laminin released by MDCK cells (O'Brien et al., 2001; Yu et al., 2005). At the same time, Rac1 is still crucial in MDCK cells embedded in the laminin‐rich Matrigel. Induced expression of dominant negative Rac1 (T17N) leads to the formation of MDCK cell clusters with inverted polarity in both collagen I and Matrigel environments (Myllymäki et al., 2011). Consistent with this, depletion of Rac1 leads to inversion of apico‐basal polarity not only in cell doublets (Figure 2) but also at later stages (Figure 3) in Matrigel‐cultured MDCK cells. Furthermore, it has been reported that in 3D Matrigel culture, MDCK cells depleted of the small GTPase Arf6 form an inverted cyst, whose inversion is restored by epidermal growth factor‐elicited Rac1 activation and by expression of a constitutively active form of Rac1 (Monteleon et al., 2012). Thus, it seems reasonable that in addition to laminin assembly, Rac1 has additional functions in polarity reorientation. They appear to include the initiation of apical protein endocytosis at the two‐cell stage, as shown in the present study. On the other hand, the requirement for Rac1 may depend on epithelial cell types. In mammary epithelial cells, β1‐integrin‐mediated signaling via integrin‐linked kinase (ILK), but not Rac1, is critical for polarized morphogenesis, which has been shown in vivo and in vitro by using cells derived from Rac1‐deleted mice (Akhtar & Streuli, 2013). The reason for the dispensability of Rac1 in mammary glands remains unknown. It might be due to the difference in the mechanisms for de novo lumen formation. 3D‐cultured MDCK cells form an apical lumen via “cord hollowing” that requires apical vesicle delivery (Iruela‐Arispe & Beitel, 2013; Jewett & Prekeris, 2018), whereas in mammary gland development, luminal space is created via “cavitation” by apoptosis of cells inside the gland (Iruela‐Arispe & Beitel, 2013; Mailleux et al., 2008); with apoptosis as the mechanism, the role of membrane trafficking, for example, Rac1‐dependent apical protein endocytosis, may be less significant. It might also be possible that Rac3, a closely‐related Rac1 homolog that is expressed in mammary epithelial cells (Leung et al., 2003; Mira et al., 2000), plays a redundant or alternative role.

In 3D Matrigel culture of mouse mammary epithelial cells, β1‐integrin and ILK are required not only for the endocytic removal of apical proteins but also for proper positioning of the Golgi apparatus (Akhtar & Streuli, 2013). By contrast, in MDCK cells, Rac1 plays a crucial role in plasma membrane polarity but provides a minor contribution to internal organelle polarity (Figure 3). Thus, Rac1 seems to be specialized for plasma membrane reorientation, which may include its importance in the initiation of apical protein endocytosis at the two‐cell stage.

Cdc42 serves at multiple stages for epithelial cystogenesis. In polarity reorientation, Cdc42 is crucial for apical vesicle exocytosis to the cell–cell contact site for PAP formation at the two‐cell stage after the first cell division (Figure 4) (Bryant et al., 2010). At later stages of cystogenesis, Cdc42 contributes to proper orientation of the mitotic spindle during metaphase of a second or later division round (Jaffe et al., 2008); Cdc42 depletion causes mitotic spindle misorientation, resulting in multiple non‐centrally located apical lumens (Jaffe et al., 2008; Martin‐Belmonte et al., 2007). Proper spindle orientation also requires two Cdc42‐specific GEFs, Tuba and Intersectin‐2, supporting a critical role of Cdc42 (Qin et al., 2010; Rodriguez‐Fraticelli et al., 2010). In addition, depletion of IQGAP1 causes mitotic spindle misorientation and multiple lumen formation (Bañón‐Rodríguez et al., 2014). This raises the possibility that IQGAP1 may serve as an effector of Cdc42 in mitotic spindle orientation. It is thus tempting to postulate that at the two‐cell stage in epithelial cystogenesis, IQGAP1 functions as a component of the Rac1‐centered complex in polarity reorientation, and at later stages, this scaffold protein in turn forms a complex with Cdc42 to orient the mitotic spindle for proper lumen formation.

For correct cystogenesis of MDCK cells, inhibition of the RhoA–ROCK1 pathway is also known to be important (Bryant et al., 2014; Yu et al., 2008). In collagen I culture, the inversion of polarity caused by β1‐integrin blockade is almost completely rescued by knockdown of RhoA and by Y27632, a ROCK kinase inhibitor; this agent also reverts the Rac1 (T17N)‐induced polarity inversion, but to a slightly lesser extent (Yu et al., 2008). In Matrigel culture, polarity inversion by β1‐integrin blockade is partially restored by depletion of RhoA or ROCK1, albeit the role of Rac1 has not been tested (Bryant et al., 2014). On the basis of the finding in collagen I culture (Yu et al., 2008), Rac1 is considered to suppress the RhoA–ROCK1 pathway via the antagonism between the Rac1 and RhoA GTPases (Lawson & Burridge, 2014). The antagonism, however, cannot fully explain the mechanism whereby Rac1 critically functions in β1‐integrin‐triggerd polarity reorientation because depletion of RhoA only partially rescued polarity misorientation in Matrigel culture (Bryant et al., 2014). Thus, Rac1 appears to reorient the polarity at the two‐cell stage via three pathways: laminin assembly (O'Brien et al., 2001; Yu et al., 2005); the Rac1–RhoA antagonism (Bryant et al., 2014; Yu et al., 2008); and the Rac1–Tiam1–IQGAP1 complex for apical protein endocytosis from the cell–ECM interface, as shown in the present study.

## 4 EXPERIMENTAL PROCEDURES

### 4.1 Plasmids

The cDNAs encoding human Rac1 and Cdc42 were prepared as previously described (Hayase et al., 2013; Noda et al., 2001); the cDNAs encoding human IQGAP1 (amino acids 1–1657) and human Tiam1 (amino acids 1–1591) were prepared by polymerase chain reaction (PCR) using Human Multiple Tissue cDNA panels (Takara Bio Inc.); and the cDNA for canine EPS15R (amino acids 1–910) was obtained by reverse transcription polymerase chain reaction using RNAs prepared from MDCK II cells. Mutations leading to the indicated amino acid substitutions were introduced by PCR‐mediated site‐directed mutagenesis. The cDNAs were ligated to the following mammalian expression vectors: pEF‐BOS (Mizushima & Nagata, 1990) or pcDNA3 (Thermo Fisher Scientific) for expression of FLAG‐, Myc‐, or HA‐tagged proteins; pEGFP‐C1 or pEGFP‐N1 (Takara Bio Inc.) for expression of N‐ or C‐terminally GFP‐tagged proteins, respectively. All of the constructs were sequenced for confirmation of their identities.

### 4.2 Antibodies

The anti‐gp135 (3F2) monoclonal antibody (Ojakian & Schwimmer, 1994) was generously gifted from Dr. G. K. Ojakian (State University of New York, USA). The anti‐FLAG (M2; #F3165) and anti‐β‐tubulin (TUB 2.1; #T4026) mouse monoclonal antibodies were purchased from Sigma‐Aldrich; the anti‐Rac1 (clone 102; #610651), anti‐Cdc42 (clone 44; #610929), anti‐β‐catenin (clone 14; #610154), and anti‐GM130 (clone 35; #610822) mouse monoclonal antibodies from BD Transduction Laboratory; the anti‐β‐catenin (H‐102; #sc‐7199), anti‐Tiam1 (C‐16; #sc‐872), and anti‐IQGAP1 (H‐109; #sc‐10792) rabbit polyclonal antibodies and the anti‐AP2α mouse monoclonal antibody (C‐8; #sc‐17771) from Santa Cruz Biotechnology; the anti‐AP2β rabbit monoclonal antibody (EPR7567; #ab129168) from Abcam; the anti‐Myc mouse monoclonal antibody (9E10; #11667203001) from Roche Applied Science; the anti‐HA mouse monoclonal antibody (16B12; #MMS‐101P) from Covance; the anti‐phospho‐ezrin (Thr567)/radixin (Thr564)/moesin (Thr558) (p‐ERM) rabbit monoclonal antibody (48G2; #3726) from Cell Signaling Technology; control IgG1 (#X0931) from Dako Cytomation; the anti‐β1‐integrin monoclonal antibody (AIIB2) from Developmental Studies Hybridoma Bank; and the mouse monoclonal antibody against green fluorescent protein (GF200; #04363‐24) from Nacalai Tesque.

### 4.3 Cell culture

MDCK II cells and HEK293T cells were cultured in Eagle's minimal essential medium (MEM) with 10% fetal calf serum (FCS) and in Dulbecco's modified Eagle's medium (DMEM) with 10% FCS, respectively.

### 4.4 Co‐immunoprecipitation analysis

Immunoprecipitation was performed as previously described (Kamakura et al., 2024; Kohda et al., 2024). Briefly, HEK293T cells were transfected using X‐tremeGENE HP DNA Transfection Reagent (Roche) with the indicated cDNAs and cultured for 48 h. For analysis of interaction between FLAG–IQGAP1 and endogenous AP2α, GFP–EPS15R was also expressed in HEK293 cells. Cells were then lysed with a lysis buffer (150 mM NaCl, 5 mM ethylenediaminetetraacetic acid (EDTA), 1 mM dithiothreitol (DTT), 0.1% Triton X‐100, 10% glycerol, and 50 mM Tris–HCl, pH 7.5) containing Protease Inhibitor Cocktail (Sigma‐Aldrich). Proteins in the lysates were precipitated with the anti‐FLAG (M2) antibody, anti‐Myc (9E10) antibody, or control IgG1, coupled to protein G‐Sepharose (GE Healthcare Biosciences). The precipitants were analyzed by immunoblot with the indicated antibodies, and the blots were developed using ImmunoStar Zeta or ImmunoStar LD (FUJIFILM Wako) for visualization.

### 4.5 Knockdown with siRNA

Double strand siRNAs targeting canine Rac1, Cdc42, IQGAP1, Tiam1, and AP2, which contain the following sequences on the sense strand of 25‐nucleotide modified synthetic RNAs (Stealth™ RNAi; Thermo Fisher Scientific), were used: Rac1 siRNA‐1, 5′‐CACAACCAAUGCAUUUCCUGGAGAA‐3′; Rac1 siRNA‐2, 5′‐ACAAAGACACGAUUGAGAAACUGAA‐3′; Cdc42 siRNA‐1, 5′‐CCACUGUCCAAAGACUCCUUUCUUG‐3′; Cdc42 siRNA‐2, 5′‐GGACCCAAAUUGAUCUCCGAGAUGA‐3′; IQGAP1 siRNA‐1, 5′‐UGGAUGAGAUUGGAUUGCCUAAGAU‐3′; IQGAP1 siRNA‐2, 5′‐CAUGCACUCAGUUUGUACCUGUUCA‐3′; Tiam1 siRNA‐1, 5′‐CAGCCUGGAAUUCUCACUCUCUGAU‐3′; Tiam1 siRNA‐2, 5′‐CAGAGCGCACCUACGUGAAAGACUU‐3′; AP2β siRNA‐1, 5′‐CACCUGGUGGAUAUGUGGCUCCUAA‐3′; AP2β siRNA‐2, 5′‐GAGCAAUCUGCAGAGCGCUGUGUAA‐3′. Medium GC Duplex of Stealth™ RNAi Negative Control Duplexes #2 (Thermo Fisher Scientific) was used as a negative control. MDCKII cells plated at 3 × 10^4^/cm^2^ were transfected with siRNA (4.8 pmol) using Lipofectamine™ RNAi MAX (Thermo Fisher Scientific) and cultured for 24 h before use in further experiments.

### 4.6 Cystogenesis and immunofluorescence microscopy

Immunofluorescence microscopy was performed as previously described (Chishiki et al., 2017; Hayase et al., 2013). For 3D culture, MDCKII cells transfected with the indicated RNA were subcultured in MEM with 10% FCS for 24 h and trypsinized to a single‐cell suspension at 1.2 × 10^4^ cells/mL in 5% Matrigel, containing laminin, type IV collagen, and entactin (Corning Costar); and 250 μL of the suspension was plated in an eight‐well cover glass chamber (Iwaki) precoated with 40 μL of Matrigel. The cells were then cultured in Matrigel for 24 h (for analysis of cysts at the two‐cell stage) or 48 h (for analysis of cysts comprising four to eight cells) before fixation. In the experiments for blockade of Matrigel‐induced integrin activation, the monoclonal antibody against β1 integrin (AIIB2) was added to the suspension medium at a final concentration of 8 μg/mL. For staining of gp135 and β‐catenin, MDCKII cells cultured in Matrigel were fixed for 30 min in 4% paraformaldehyde and subsequently permeabilized for 30 min in phosphate‐buffered saline (PBS; 137 mM NaCl, 2.7 mM KCl, 8.1 mM Na_2_HPO_4_, and 1.5 mM KH_2_PO_4_, pH 7.4) containing 0.5% Triton X‐100 and 3% bovine serum albumin (BSA). For Rac1 staining, cells were fixed for exactly 10 min in 2% formaldehyde and permeabilized for 30 min in PBS containing 0.5% Triton X‐100 and 3% BSA. For staining of GM130, cells were fixed for 2 min in 100% methanol at 4°C and then for 10 min in 4% paraformaldehyde, followed by blocking for 30 min with PBS containing 3% BSA. For staining of IQGAP1, cells were fixed for 10 min in 4% paraformaldehyde, permeabilized for 30 min in PBS containing 0.5% Triton X‐100 and 3% BSA, and then incubated with the primary antibody for 20 days. Indirect immunofluorescence analysis was performed using the following secondary antibodies: Alexa Fluor 488‐labeled goat anti‐rabbit or anti‐mouse IgG antibodies; Alexa Fluor 594‐labeled anti‐rabbit or anti‐mouse IgG antibodies (Thermo Fisher Scientific). Actin filaments were stained with Alexa Fluor 647 phalloidin (Thermo Fisher Scientific) and nuclei with Hoechst 33342 (Thermo Fisher Scientific). Confocal images were captured at room temperature on the confocal microscope LSM780 (Carl Zeiss) and analyzed using ZEN software (Carl Zeiss). The microscopes were equipped with a Plan‐Apochromat 40×/1.3 NA oil immersion objective lens.

For analysis of cyst morphogenesis at the two‐cell stage, MDCKII cells were grown for 24 h in 3D Matrigel culture. The doublet with “fully‐inverted” or “partially‐inverted” polarity was defined as that with gp135 distributed to more or less than 75% of the ECM‐abutting plasma membranes, respectively. The doublet with apical vesicles or with a PAP was defined as those containing apical vesicles in the cytoplasm or PAP on the cell–cell contact, respectively, with β‐catenin distributed over the whole ECM‐abutting plasma membrane.

For analysis of cysts comprising four to eight cells, MDCKII cells were grown for 48 h in Matrigel. Normal cysts had a single lumen surrounded by cells that exhibit both intense gp135 staining at the apical surface and β‐catenin staining at the surface facing the ECM. Cysts with gp135 at the surface facing the ECM were designated as cysts with inverted orientation, and cysts containing more than two lumens with gp135 staining were designated as cysts with multiple lumens. To discriminate lumens from cytoplasmic gp135‐rich vesicles/vacuoles, we defined a lumen as a gp135‐rich structure that makes contact with β‐catenin‐containing membranes. We tested more than 75 doublets or cysts for the analysis of their morphology.

### 4.7 Protein identification by liquid chromatography–tandem mass spectrometry analysis

For identifying effectors of Rac1, MDCK cells were transfected with a plasmid vector encoding FLAG‐tagged Rac1 (Q61L) or Rac1 (T17N) or with an empty FLAG vector. For exploring the binding partners of IQGAP1, HEK293T cells were transfected with a plasmid vector encoding FLAG–IQGAP1 or with the empty vector. The transfected cells were cultured for 48 h and then lysed at 4C with a lysis buffer (150 mM NaCl, 5 mM EDTA, 1 mM DTT, 0.5% Triton X‐100, 10% glycerol, and 50 mM Tris–HCl, pH 7.5) containing Protease Inhibitor Cocktail (Sigma‐Aldrich). Proteins in the lysates were precipitated with the anti‐FLAG (M2) antibody‐conjugated magnetic beads (Sigma‐Aldrich). After washing three times with the lysis buffer, the proteins were subjected to SDS–PAGE, followed by silver staining. Protein identification using LC–MS/MS was performed at the Laboratory for Research Support, Medical Institute of Bioregulation, Kyushu University, according to the protocol of Matsumoto et al. (2017). Bands separated by SDS–PAGE were excised from the gel, and the proteins in the gel were digested with trypsin gold mass spectrometry grade (PROMEGA) dissolved in 25 mM ammonium bicarbonate solution. The gel‐extracted peptides were subjected to nano‐LC–MS/MS analysis using an LTQ Orbitrap Velos Pro mass spectrometer system (Thermo Fisher Scientific) coupled with an Advance UHPLC system (Bruker) and an HTC‐PAL autosampler (CTC Analytics). MS/MS spectra were obtained automatically in a data‐dependent scan mode and compared with those in the UniProtKB/SwissProt human peptide database (UniProt Consortium) using the MASCOT search engine (Matrix Science). Assigned high‐scoring peptide sequences were manually confirmed by comparison with the corresponding spectra.

### 4.8 Statistical analysis

Statistical differences were analyzed by one‐way analysis of variance with Tukey–Kramer's multiple comparison of means test.

## AUTHOR CONTRIBUTIONS

**Michihiro Horikawa:** Conceptualization; data curation; formal analysis; investigation; methodology; visualization; writing – original draft; writing – review and editing. **Junya Hayase:** Conceptualization; data curation; formal analysis; funding acquisition; investigation; methodology; resources; supervision; visualization; writing – original draft; writing – review and editing. **Sachiko Kamakura:** Conceptualization; methodology; data curation; supervision; resources; formal analysis; visualization; writing – review and editing; writing – original draft; funding acquisition. **Akira Kohda:** Data curation; formal analysis; resources; supervision; writing – review and editing. **Masafumi Nakamura:** Supervision; writing – review and editing. **Hideki Sumimoto:** Conceptualization; data curation; formal analysis; funding acquisition; methodology; project administration; resources; supervision; writing – original draft; writing – review and editing.

## CONFLICT OF INTEREST STATEMENT

The authors have no conflict of interest.

## Data Availability

Data sharing not applicable to this article as no datasets were generated or analysed during the current study.

## References

[gtc13169-bib-0001] Akhtar, N. , & Streuli, C. H. (2013). An integrin‐ILK‐microtubule network orients cell polarity and lumen formation in glandular epithelium. Nature Cell Biology, 15(1), 17–27. 10.1038/ncb2646 23263281 PMC3701152

[gtc13169-bib-0002] Bañón‐Rodríguez, I. , Gálvez‐Santisteban, M. , Vergarajauregui, S. , Bosch, M. , Borreguero‐Pascual, A. , & Martín‐Belmonte, F. (2014). EGFR controls IQGAP basolateral membrane localization and mitotic spindle orientation during epithelial morphogenesis. The EMBO Journal, 33(2), 129–145. 10.1002/embj.201385946 24421325 PMC3989607

[gtc13169-bib-0003] Beacham, G. M. , Partlow, E. A. , & Hollopeter, G. (2019). Conformational regulation of AP1 and AP2 clathrin adaptor complexes. Traffic, 20(10), 741–751. 10.1111/tra.12677 31313456 PMC6774827

[gtc13169-bib-0004] Bernascone, I. , Hachimi, M. , & Martin‐Belmonte, F. (2017). Signaling networks in epithelial tube formation. Cold Spring Harbor Perspectives in Biology, 9(12), a027946. 10.1101/cshperspect.a027946 28246178 PMC5710102

[gtc13169-bib-0005] Bryant, D. M. , Datta, A. , Rodríguez‐Fraticelli, A. E. , Peränen, J. , Martín‐Belmonte, F. , & Mostov, K. E. (2010). A molecular network for *de novo* generation of the apical surface and lumen. Nature Cell Biology, 12(11), 1035–1045. 10.1038/ncb2106 20890297 PMC2975675

[gtc13169-bib-0006] Bryant, D. M. , Roignot, J. , Datta, A. , Overeem, A. W. , Kim, M. , Yu, W. , Peng, X. , Eastburn, D. J. , Ewald, A. J. , Werb, Z. , & Mostov, K. E. (2014). A molecular switch for the orientation of epithelial cell polarization. Developmental Cell, 31(2), 171–187. 10.1016/j.devcel.2014.08.027 25307480 PMC4248238

[gtc13169-bib-0007] Buckley, C. E. , & St Johnston, D. (2022). Apical‐basal polarity and the control of epithelial form and function. Nature Reviews Molecular Cell Biology, 23(8), 559–577. 10.1038/s41580-022-00465-y 35440694

[gtc13169-bib-0008] Chishiki, K. , Kamakura, S. , Hayase, J. , & Sumimoto, H. (2017). Ric‐8A, an activator protein of Gαi, controls mammalian epithelial cell polarity for tight junction assembly and cystogenesis. Genes to Cells, 22(3), 293–309. 10.1111/gtc.12477 28185378

[gtc13169-bib-0009] Dixon, E. E. , & Woodward, O. M. (2018). Three‐dimensional in vitro models answer the right questions in ADPKD cystogenesis. American Journal of Physiology. Renal Physiology, 315(2), F332–F335. 10.1152/ajprenal.00126.2018 29693448 PMC6334996

[gtc13169-bib-0010] Ferrari, A. , Veligodskiy, A. , Berge, U. , Lucas, M. S. , & Kroschewski, R. (2008). ROCK‐mediated contractility, tight junctions and channels contribute to the conversion of a preapical patch into apical surface during isochoric lumen initiation. Journal of Cell Science, 121(21), 3649–3663. 10.1242/jcs.018648 18946028

[gtc13169-bib-0011] Hayase, J. , Kamakura, S. , Iwakiri, Y. , Yamaguchi, Y. , Izaki, T. , Ito, T. , & Sumimoto, H. (2013). The WD40 protein Morg1 facilitates Par6–aPKC binding to Crb3 for apical identity in epithelial cells. Journal of Cell Biology, 200(5), 635–650. 10.1083/jcb.201208150 23439680 PMC3587828

[gtc13169-bib-0012] Iruela‐Arispe, M. L. , & Beitel, G. J. (2013). Tubulogenesis. Development, 140(14), 2851–2855. 10.1242/dev.070680 23821032 PMC3699276

[gtc13169-bib-0013] Jaffe, A. B. , Kaji, N. , Durgan, J. , & Hall, A. (2008). Cdc42 controls spindle orientation to position the apical surface during epithelial morphogenesis. Journal of Cell Biology, 183(4), 625–633. 10.1083/jcb.200807121 19001128 PMC2582895

[gtc13169-bib-0014] Jewett, C. E. , & Prekeris, R. (2018). Insane in the apical membrane: Trafficking events mediating apicobasal epithelial polarity during tube morphogenesis. Traffic, 19(9), 666–678. 10.1111/tra.12579 PMC6239989 29766620

[gtc13169-bib-0015] Kamakura, S. , Hayase, J. , Kohda, A. , Iwakiri, Y. , Chishiki, K. , Izaki, T. , & Sumimoto, H. (2024). TMEM25 is a Par3‐binding protein that attenuates claudin assembly during tight junction development. EMBO Reports, 25(1), 144–167. 10.1038/s44319-023-00018-0 38177906 PMC10897455

[gtc13169-bib-0016] Karnoub, A. E. , Worthylake, D. K. , Rossman, K. L. , Pruitt, W. M. , Campbell, S. L. , Sondek, J. , & Der, C. J. (2001). Molecular basis for Rac1 recognition by guanine nucleotide exchange factors. Nature Structural Biology, 8(12), 1037–1041. 10.1038/nsb719 11685227

[gtc13169-bib-0017] Kohda, A. , Kamakura, S. , Hayase, J. , & Sumimoto, H. (2024). The NADPH oxidases DUOX1 and DUOX2 are sorted to the apical plasma membrane in epithelial cells via their respective maturation factors DUOXA1 and DUOXA2. Genes to Cells 29(10), 921–930. 10.1111/gtc.13153 PMC11555622 39126279

[gtc13169-bib-0018] Lawson, C. D. , & Burridge, K. (2014). The on‐off relationship of rho and Rac during integrin‐mediated adhesion and cell migration. Small GTPases, 5(1), e27958. 10.4161/sgtp.27958 24607953 PMC4114617

[gtc13169-bib-0019] Leung, K. , Nagy, A. , Gonzalez‐Gomez, I. , Groffen, J. , Heisterkamp, N. , & Kaartinen, V. (2003). Targeted expression of activated Rac3 in mammary epithelium leads to defective postlactational involution and benign mammary gland lesions. Cells, Tissues, Organs, 175(2), 72–83. 10.1159/000073751 14605486

[gtc13169-bib-0020] Lubarsky, B. , & Krasnow, M. A. (2003). Tube morphogenesis: Making and shaping biological tubes. Cell, 112(1), 19–28. 10.1016/S0092-8674(02)01283-7 12526790

[gtc13169-bib-0021] Mailleux, A. A. , Overholtzer, M. , & Brugge, J. S. (2008). Lumen formation during mammary epithelial morphogenesis: Insights from in vitro and in vivo models. Cell Cycle, 7(1), 57–62. 10.4161/cc.7.1.5150 18196964

[gtc13169-bib-0022] Marei, H. , Carpy, A. , Macek, B. , & Malliri, A. (2016). Proteomic analysis of Rac1 signaling regulation by guanine nucleotide exchange factors. Cell Cycle, 15(15), 1961–1974. 10.1080/15384101.2016.1183852 27152953 PMC4968972

[gtc13169-bib-0023] Martin‐Belmonte, F. , Gassama, A. , Datta, A. , Yu, W. , Rescher, U. , Gerke, V. , & Mostov, K. (2007). PTEN‐mediated apical segregation of phosphoinositides controls epithelial morphogenesis through Cdc42. Cell, 128(2), 383–397. 10.1016/j.cell.2006.11.051 17254974 PMC1865103

[gtc13169-bib-0024] Matlin, K. S. , Myllymäki, S. M. , & Manninen, A. (2017). Laminins in epithelial cell polarization: Old questions in search of new answers. Cold Spring Harbor Perspectives in Biology, 9(10), a027920. 10.1101/cshperspect.a027920 28159878 PMC5629996

[gtc13169-bib-0025] Matsumoto, M. , Matsuzaki, F. , Oshikawa, K. , Goshima, N. , Mori, M. , Kawamura, Y. , Ogawa, K. , Fukuda, E. , Nakatsumi, H. , Natsume, T. , Fukui, K. , Horimoto, K. , Nagashima, T. , Funayama, R. , Nakayama, K. , & Nakayama, K. I. (2017). A large‐scale targeted proteomics assay resource based on an in vitro human proteome. Nature Methods, 14(3), 251–258. 10.1038/nmeth.4116 28267743

[gtc13169-bib-0026] Mettlen, M. , Chen, P. H. , Srinivasan, S. , Danuser, G. , & Schmid, S. L. (2018). Regulation of clathrin‐mediated endocytosis. Annual Review of Biochemistry, 87(1), 871–896. 10.1146/annurev-biochem-062917-012644 PMC6383209 29661000

[gtc13169-bib-0027] Mira, J. P. , Benard, V. , Groffen, J. , Sanders, L. C. , & Knaus, U. G. (2000). Endogenous, hyperactive Rac3 controls proliferation of breast cancer cells by a p21‐activated kinase‐dependent pathway. Proceedings of the National Academy of Sciences of the United States America, 97(1), 185–189. 10.1073/pnas.97.1.185 PMC26637 10618392

[gtc13169-bib-0028] Mizushima, S. , & Nagata, S. (1990). pEF‐BOS, a powerful mammalian expression vector. Nucleic Acids Research, 18(17), 5322. 10.1093/nar/18.17.5322 1698283 PMC332193

[gtc13169-bib-0029] Monteleon, C. L. , Sedgwick, A. , Hartsell, A. , Dai, M. , Whittington, C. , Voytik‐Harbin, S. , & D'Souza‐Schorey, C. (2012). Establishing epithelial glandular polarity: Interlinked roles for ARF6, Rac1, and the matrix microenvironment. Molecular Biology of the Cell, 23(23), 4495–4505. 10.1091/mbc.E12-03-0246 23051733 PMC3510012

[gtc13169-bib-0030] Mrozowska, P. S. , & Fukuda, M. (2016). Regulation of podocalyxin trafficking by Rab small GTPases in 2D and 3D epithelial cell cultures. Journal of Cell Biology, 213(3), 355–369. 10.1083/jcb.201512024 27138252 PMC4862332

[gtc13169-bib-0031] Myllymäki, S. M. , Teräväinen, T. P. , & Manninen, A. (2011). Two distinct integrin‐mediated mechanisms contribute to apical lumen formation in epithelial cells. PLoS One, 6(5), e19453. 10.1371/journal.pone.0019453 21573123 PMC3089628

[gtc13169-bib-0032] Noda, Y. , Takeya, R. , Ohno, S. , Naito, S. , Ito, T. , & Sumimoto, H. (2001). Human homologues of the *Caenorhabditis elegans* cell polarity protein PAR6 as an adaptor that links the small GTPases Rac and Cdc42 to atypical protein kinase C. Genes to Cells, 6(2), 107–119. 10.1046/j.1365-2443.2001.00404.x 11260256

[gtc13169-bib-0033] O'Brien, L. E. , Jou, T. S. , Pollack, A. L. , Zhang, Q. , Hansen, S. H. , Yurchenco, P. , & Mostov, K. E. (2001). Rac1 orientates epithelial apical polarity through effects on basolateral laminin assembly. Nature Cell Biology, 3(9), 831–838. 10.1038/ncb0901-831 11533663

[gtc13169-bib-0034] Ojakian, G. K. , & Schwimmer, R. (1994). Regulation of epithelial cell surface polarity reversal by β1 integrins. Journal of Cell Science, 107(3), 561–576. 10.1242/jcs.107.3.561 7516342

[gtc13169-bib-0035] Qin, Y. , Meisen, W. H. , Hao, Y. , & Macara, I. G. (2010). Tuba, a Cdc42 GEF, is required for polarized spindle orientation during epithelial cyst formation. Journal of Cell Biology, 189(4), 661–669. 10.1083/jcb.201002097 20479467 PMC2872902

[gtc13169-bib-0036] Rodriguez‐Fraticelli, A. E. , Vergarajauregui, S. , Eastburn, D. J. , Datta, A. , Alonso, M. A. , Mostov, K. , & Martín‐Belmonte, F. (2010). The Cdc42 GEF Intersectin 2 controls mitotic spindle orientation to form the lumen during epithelial morphogenesis. Journal of Cell Biology, 189(4), 725–738. 10.1083/jcb.201002047 20479469 PMC2872911

[gtc13169-bib-0037] Román‐Fernández, A. , & Bryant, D. M. (2016). Complex polarity: Building multicellular tissues through apical membrane traffic. Traffic, 17(12), 1244–1261. 10.1111/tra.12417 27281121

[gtc13169-bib-0038] Thines, L. , Roushar, F. J. , Hedman, A. C. , & Sacks, D. B. (2023). The IQGAP scaffolds: Critical nodes bridging receptor activation to cellular signaling. Journal of Cell Biology, 222(6), e202205062. 10.1083/jcb.202205062 37071417 PMC10120595

[gtc13169-bib-0039] Usatyuk, P. V. , Gorshkova, I. A. , He, D. , Zhao, Y. , Kalari, S. K. , Garcia, J. G. , & Natarajan, V. (2009). Phospholipase D‐mediated activation of IQGAP1 through Rac1 regulates hyperoxia‐induced p47^ *phox* ^ translocation and reactive oxygen species generation in lung endothelial cells. Journal of Biological Chemistry, 284(22), 15339–15352. 10.1074/jbc.M109.005439 19366706 PMC2685714

[gtc13169-bib-0040] Yu, W. , Datta, A. , Leroy, P. , O'Brien, L. E. , Mak, G. , Jou, T. S. , Matlin, K. S. , Mostov, K. E. , & Zegers, M. M. P. (2005). β1‐integrin orients epithelial polarity via Rac1 and laminin. Molecular Biology of the Cell, 16(2), 433–445. 10.1091/mbc.e04-05-0435 15574881 PMC545874

[gtc13169-bib-0041] Yu, W. , Shewan, A. M. , Brakeman, P. , Eastburn, D. J. , Datta, A. , Bryant, D. M. , Fan, Q.‐W. , Weiss, W. A. , Zegers, M. M. P. , & Mostov, K. E. (2008). Involvement of RhoA, ROCK I and myosin II in inverted orientation of epithelial polarity. EMBO Reports, 9(9), 923–929. 10.1038/embor.2008.135 18660750 PMC2529350

